# Transfert et transport des nouveau-nés en situation de détresse vitale à Yaoundé, Cameroun: analyse situationnelle dans un hôpital de référence

**DOI:** 10.11604/pamj.2016.25.214.9642

**Published:** 2016-12-06

**Authors:** Anne Esther Njom Nlend, Cécile Zeudja, Lydie Nsoa

**Affiliations:** 1 Centre Hospitalier d’Essos, Caisse Nationale de Prévoyance Sociale, Service de Pédiatrie et de Prévention Infantile, BP 5777 Yaoundé, Cameroun

**Keywords:** Nouveau-né, transport, référence, Newborns, transport, reference

## Abstract

**Introduction:**

Notre objectif était de décrire les caractéristiques du transfert des nouveau-nés en situation de détresse vitale vers le Centre Hospitalier d’ESSOS à Yaoundé, Cameroun.

**Méthodes:**

Nous avons réalisé une étude transversale prospective d’Octobre 2014 à Janvier 2015. La collecte des données était faite à l’aide d’un questionnaire court auprès de l’opérateur du transfert. Principaux paramètres: moyen de transport, motif du transfert, délai du transfert, nombre de décours (itinéraire avant l’admission) prévalence de l’hypothermie, taux de mortalité néonatale.

**Résultats:**

Nous avons enregistré 73 transferts durant la période Près de 1/5 (22%) étaient nés au sein du district de santé de la structure de référence. Les nouveau-nés référés venaient de structures tertiaires dans 24/73 (33%). Le principal motif de transfert était la grande prématurité (40%) suivi de l’asphyxie néonatale (26%). Le transfert était médicalisé dans 5/73 (7%) des cas et infirmier interhospitalier dans 10/73 (13,6%) des cas. Le délai moyen de transfert était de 17 heures, 60% avaient été transférés dans les 6 premières heures de vie et 22% (16/73) dans les deux heures. Pour plus de la moitié des nouveau-nés, un détour dans une autre structure avait été réalisé avant l’admission. L’hypothermie (température centrale inférieure à 36°) à l’arrivée était notée dans 20% des cas. 15/73(20,5%) des nouveau-nés transférés sont décédés. la température moyenne à l’arrivée des nouveau-nés décédés étaient de 35°5C vs 37°C chez les non-décédés (p=0,006). Le pourcentage de nouveau-nés ayant subi plus de 2 détours était de 57% chez les décédés versus 30% chez les non-décédés (p=0,02).

**Conclusion:**

A Yaoundé, les conditions de transferts des nouveau-nés précaires grèvent le pronostic néonatal immédiat à cause d’un itinéraire erratique qui majore le risque d’hypothermie et de décès, ceci renforce la nécessité de mise en place d’un réseau périnatal.

## Abstract (en)

**Introduction:**

This study aims to describe transfer modalities of newborn babies in vital distress to the ESSOS Hospital Center in Yaoundé, Cameroon.

**Methods:**

We conducted a prospective cross-sectional study from October 2014 to January 2015. Data were collected using a short questionnaire from the transfer operator. Main parameters: means of transport, reason for transfer, transfer delay, number of detours (itinerary before admission) prevalence for hypothermia, neonatal mortality rate.

**Results:**

We recorded 73 transfers during the study period. Nearly 1/5 (22%) of infants were born within the health district of the reference structure. 24/73 newborns were referred for tertiary care centers (33%). The main reason for transfer was prematurity (40%) followed by neonatal asphyxia (26%). Medical transfer was performed in 5/73 (7%) cases, inter-hospital transfer effected through a nurse was performed in 10/73 (13.6%) cases. The average transfer time was 17 hours, 60% of babies were transferred within the first 6 hours of life, 22% (16/73) within the first two hours of life. For more than half of newborns, a transfer to another hospital was done before admission. Hypothermia (central temperature less than 36° C) on arrival was found in 20% of cases. 15/73 (20.5%) of transferred newborns died. The mean temperature in the dead infants upon their arrival to the hospital was 35.5° C versus 37° C in the non-deceased (p = 0.006). The percentage of newborns who underwent =2 tranfers was 57% in the deceased infants versus 30% in the non-deceased ones (p=0,02).

**Conclusion:**

In Yaoundé, transfers conditions of newborns in precarious conditions of life hinder early neonatal prognosis because of an erratic itinerary, which increases the risk of hypothermia and death. This reinforces the need for a perinatal network.

## Introduction

En 2014, il était estimé qu’environ 2,8 millions de nouveau-nés étaient décédés durant les 28 premiers jours de vie dans le monde, la plupart en Afrique Subsaharienne [1]. Si les causes de ces décès néonatals sont largement connues, les autres données environnementales du système de santé relatives à la faiblesse de l’organisation des soins et au réseautage sont moins rapportées. L’effectivité des réseaux périnatals a été prouvée pour améliorer le parcours et l’itinéraire des femmes enceintes et de leurs nouveau-nés, notamment dans les situations à risque [2, 3]. Ces réseaux sont le plus souvent institutionnels ou spontanés et concourent à définir le niveau optimal de soins attendu par niveau de la pyramide sanitaire. Ils permettent également d’améliorer le transport et le transfert du nouveau-né en détresse tout en privilégiant le transfert in utero [4, 5]. Le Cameroun est un pays à fort taux de natalité avec près de 875000 naissances par an et un taux de mortalité néonatale élevée [6]. Le dispositif institutionnel qui définit le paquet de service par niveau de formation sanitaire n’est pas spécifique au nouveau-né [7]; de plus, parmi les structures qui abritent une maternité, le pole d’accueil du nouveau-né est souvent incomplet. Cette insuffisance explique que les références soient souvent faites à la naissance après constat d’anomalies en salle de travail. Au niveau de la ville de Yaoundé qui couvre une superficie de 180 km2 avec 6 districts de santé, les structures équipées pour accueillir les nouveau-nés en situation de détresse vitale sont insuffisantes [7]. La communication et le transfert entre les structures sanitaires de premier niveau et celles de niveau plus élevé n’est pas organisée. Le transport des nouveau-nés en détresse est empirique et l’accès dans la structure référente assujettie aux conditions de coûts, en l’absence de couverture médicale universelle [8]. Au regard de ce qui précède, nous avons voulu, en tant que pôle de référence effectuer une analyse des conditions de transport et transfert intra-urbain des nouveau-nés en détresse à Yaoundé. Cette démarche s’inscrivait dans la perspective de démarrer les activités d’un réseau périnatal à Yaoundé pour contribuer à la réduction de la mortalité néonatale [9]. L’objectif de notre étude était de décrire les délais de transfert des nouveau-nés en situation de détresse vitale vers notre structure ainsi que les principaux motifs de référence.

## Méthodes

**Type d’étude:** il s’agissait d’une étude transversale descriptive.

**Site de l’étude et période de l’étude:** le site de l’étude était le centre hospitalier d’ESSOS (CHE) de la ville de Yaoundé. Ce site est une structure sanitaire publique à gestion privée. Les soins y sont payants. Une caution d’hospitalisation d’environ 80 euros soit prés de deux fois le SMIC camerounais est requise à l’entrée. Cette structure se positionne au niveau le plus élevé de la pyramide sanitaire en matière de soins de nouveau-nés. Autour d’elle, le CHE a organisé un réseau périnatal dans le district de santé de Djoungolo autour de 25 formations sanitaires. En dehors de ce district, existe 4 autres structures équivalentes dans la ville, elles sont strictement publiques. Cette étude s’est déroulée en Juin 2015 et concernait les enfants admis entre Octobre 2014 et Janvier 2015.

**Collecte des données et analyse:** nous avons utilisé un questionnaire court pour interroger les accompagnants des nouveau-nés venant de l’extérieur et référés vers notre site. Le questionnaire court permettait d’explorer : le lieu de naissance, l’heure de naissance, l’arrivée dans le service, le durée du transfert, le nombre de détours faits avant d’arriver au CHE, la principale pathologie, la température de l’enfant à l’arrivée, l’issue immédiate (décès néonatal précoce). Etait considéré comme médicalisé tout transport impliquant une ambulance avec un personnel soignant accompagnateur et infirmier interhospitalier tout transport impliquant le personnel soignant hors ambulance. Les données descriptives ont été décrites en % et en moyenne; leur comparaison a utilisé le test exact de Fisher et le test de Student. Etait considérée comme significative toute p-value à 0,05.

**Considérations éthiques:** cette étude a obtenu la clearance du comité d’éthique institutionnel du CHE ainsi que l’autorisation administrative dudit centre.

## Résultats

Nous avons en registré 73 transferts durant la période de l’étude. Parmi les nouveau-nés transférés, 1/73 était né hors de la ville. Les 72 autres étaient nés dans Yaoundé, et près de 1/5 (22%) étaient nés au sein du district de santé de la structure de référence. La structure d’origine était de niveau tertiaire ou équivalent dans 24/73 (33 %), une structure de niveau inférieur type hôpital de district dans 15/73(20%) ou venant d’un centre de santé premier niveau de soins dans 34/73 (47 %). Le principal motif de transfert était la prématurité suivi de l’asphyxie néonatale. Le transfert essentiellement assuré par la famille, était essentiellement non médicalisé par voie terrestre. Le délai de transfert était long d’environ 17 heures; près de 40% des enfants n’avaient toujours pas été transférés dans les 6 premières heures de vie et seuls 22% étaient transférés à deux heures de vie. Ce long délai de transfert était secondaire à un détour par autre une formation sanitaire dans plus de la moitié des cas. A l’arrivée, une température inférieure à 36.5 était notée chez un nouveau-né sur 3 et près d’un nouveau-né sur 4 avait une température inférieure à 36°. Le Tableau 1 résume les caractéristiques du transfert et de la population transférée. 20% des nouveau-nés transférés vont décéder en période néonatale, la température moyenne à l’arrivée des nouveau-nés décédés étaient de 35°5C vs 37°C chez les non décédés (p=0,006), de plus, le pourcentage de nouveau-nés ayant subi plus de 2 détours était de 57% chez les décédés versus 30% chez les non décédés (p=0,02) (Tableau 2).

**Tableau 1:** Caractéristiques du transfert et des nouveau-nés transférés vers le Centre Hospitalier d’Essos à Yaoundé, Cameroun

Nouveau-nés	N(%)
Terme de naissance en semaine d'aménorrhée (moyenne, Ecart Type(ET))	35,2(4,4)
Accouchement par césarienne	17/73(17,8%)
Poids de naissance en kgs (moyenne, ET)	2,4(0,9)
Nés au sein du district	14/64(21,8%)
**Motif du transfert**	
Prématurité	29/73(40%)
Asphyxie néonatale	19/73(26%)
Fièvre néonatale	6/73(8%)
Détresse respiratoire	14/73(19%)
Autres	5/73(7%)
**Délai de transfert en heure(moyenne, ET)**	17(4,8)
Transféré dans les deux heures	15/73(20,5%)
Transféré dans les six heures	44/73(60,3%)
**Nombre de détours avant l'admission (N=56)**	
Nouveau-né ayant fait au moins un détour	30/56(53%)
Nouveau ayant fait deux détours ou plus	20/56(35%)
**Mode de transport**	
Médicalisé	10/69(14,5%)
**Température moyenne à l'arrivée en d°C**	36.7(1.1)
Nouveau-nés ayant température ≤35°	5/72(7%)
Nouveau-nés ayant température ≤36°	17/72(23%)
Nouveau-nés ayant température <36.5°	23/72(32%)
Décédés	15/73(20,5%)

**Tableau 2:** Analyse comparée des nouveau-nés décédés et non décédés après transfert

Caractéristiques des nouveau-nés	Décédés	Vivants	p-value
Température à l'arrivée en d° (moyenne)	35,5	37	0,006
Pourcentage de Transfert de plus de 6 heures	57	30	0,02
Pourcentage d’enfants ayant fait ≥deux détours (ratio)	57	30	0,02
Transfert médicalisé (ratio)	1/15(7%)	9/58(15%)	0,34

## Discussion

Ce travail descriptif a le mérite de montrer les dysfonctionnements du transfert et du transport en période néonatale dans la ville de Yaoundé. Le faille majeure identifiée est le déficit de la chaine d’information composante majeure du transfert néonatal; cette insuffisance occasionne plusieurs détours avant la structure référente finale avec pour corollaire une durée de transport trop longue nettement supérieure aux 90 minutes à deux heures recommandées pour des transferts terrestres [10, 11]. Ce délai allongé est provoqué par le personnel qui refuse d’admettre le nouveau-né à leur porte en disant ici, *“il n’y a pas de place aller à….” ou encore “ici, il a rupture d’oxygène aller à….”*, sans s’assurer au préalable que là-bas, il y ait une place et/ou que les conditions d’accueil soient remplies. Mais au-delà de ces dysfonctionnements, certaines de ces familles en détresse, sont également à la recherche de fonds pour payer les frais d’admission. Ce long délai de transfert en terrestre compromet la prise en charge appropriée et la survie néonatale précoce [12] et, sur le plan médical, une des conséquences répertoriées est le taux élevé d’hypothermie. Ce risque est connu et les taux enregistrés dans notre série sont similaires à ceux notés précédemment [13–15]. Outre le long délai de transfert, ce travail confirme la faiblesse du transport néonatal Yaoundé. Le taux de transport par ambulance de 6,8%, sensiblement plus élevé que celui rapporté en Inde (2,5%) avec un profil similaire des autres modes de transports terrestres répertoriés à savoir : taxis, véhicule personnel [16]. Ceci relève sans conteste la nécessité d’ambulance spécifique au nouveau-né voire à la mère en cas de transfert in utero à Yaoundé [17]. Toutefois, il convient de souligner la fréquence non négligeable du transport infirmier interhospitalier [18]; option qui pourrait être encouragée en l’absence d’ambulance, compte tenu de l’efficacité rapportée de tels moyens pour réduire la mortalité néonatale précoce post transfert, chez les nouveau-nés stabilisés [15, 17]. Au demeurant, cette étude n’a rien apporté de nouveau sur les principales indications de transferts qui y sont conformes à celles précédemment décrites à savoir: prématurité, asphyxie néonatale/convulsions détresse respiratoire et fièvre néonatale [11, 13]. Pour ce qui est de la mortalité observée, essentiellement précoce des nouveau-nés transférés, c’est un argument en faveur du transfert in utero qui présuppose une régionalisation correcte des soins, et un système de transport approprié [3, 4, 19]. Au total, cette étude descriptive nous a permis d’appréhender les pratiques du transfert néonatal à Yaoundé. Nous en reconnaissons les limites, notamment l’absence d’explorations des conséquences métaboliques et hypoxiques de ces longs transferts et suggérons la prise en compte de ces paramètres dans une étude ultérieure.

## Conclusion

L’organisation du transfert néonatal à Yaoundé est précaire avec des déficits notables dans la chaine de l’information et celle du chaud, ce qui renforce nécessité de mise en place d’un réseau périnatal. Recommandations : les principales recommandations qui découlent de ce travail sont la mise en place urgente d’un réseau téléphonique au sein de la ville pour réduire les itinéraires erratiques et la formalisation du transport infirmier interhospitalier.

### Etat des connaissances actuelles sur le sujet

- L’importance des réseaux en périnatalité est largement documentée pour un meilleur itinéraire thérapeutique des nouveau-nés à risque et de leur mère, notamment la sécurité lors transport et la référence autour de la naissance. Cette sécurité permet d’éviter l’hypothermie, risque majeur au cours du transport du nouveau-né.

### Contribution de notre étude à la connaissance

- Notre travail descriptif, relève les faiblesses du transport néonatal à Yaoundé et souligne l’intérêt de renforcer la chaine de communication élément essentiel pour sécuriser le transfert du nouveau-né. De plus, les carences organisationnelles suggère la mise en place d’un réseau périnatal.
